# Understanding adolescents’ perspectives on suicide risk screening in primary care: Implications and insights for implementation and practice

**DOI:** 10.1371/journal.pmen.0000607

**Published:** 2026-05-07

**Authors:** Sarah Danzo, Adia Abler, Annie Hoang, Katherine-Anne Comtois, Andrea Hartzler, Laura Richardson, Elizabeth McCauley

**Affiliations:** 1 Department of Psychiatry and Behavioral Sciences, University of Washington, Seattle, Washington, United States of America; 2 Seattle Children’s Hospital, Seattle, Washington, United States of America; 3 Department of Biomedical Informatics and Medical Education, University of Washington, Seattle, Washington, United States of America; 4 Department of Pediatrics, University of Washington, Seattle, Washington, United States of America; PLOS: Public Library of Science, UNITED KINGDOM OF GREAT BRITAIN AND NORTHERN IRELAND

## Abstract

Suicide is a pervasive public health concern with risk increasing during adolescence. Universal suicide screening for youth ages 12 years and older has been deemed an important component of suicide prevention. However, little is known about how adolescents feel about screening. This study aimed to better understand how adolescents perceive current suicide risk screening practices in primary care (PC). Purposive sampling identified adolescents with histories of suicidal thoughts or behaviors (STB) who saw a doctor in urban, suburban, or rural pediatric PC clinics at least yearly. Twenty 12–18-year-old adolescents completed semi-structured interviews exploring their views of suicide screening in PC. Interviews were recorded, transcribed, and analyzed using thematic analysis. Themes were compared by geographic setting and described across clinics as a whole. Results were similar across settings and highlight five main themes: (1) asking about STB in PC is important and shows providers care, (2) some adolescents expressed discomfort with the language used in suicide risk screeners and how questions about STB are asked, (3) provider-patient interactions and relationships can either facilitate or hinder STB disclosure, (4) mental health stigma makes disclosing STB feel uncomfortable, and (5) adolescents fear how parents/caregivers will react and potential consequences of disclosing STB (e.g., losing autonomy). Participants overwhelmingly reported that asking adolescents about STB in PC is important. However, several challenges were identified that make it difficult for adolescents to answer questions about STB honestly. Participants suggested several areas for improvement to enhance screening practices and improve adolescent disclosure.

## Introduction

Suicide is a leading cause of death among 10-to-24-year-olds in the United States [1]. To address this, several efforts have emerged to prevent adolescent suicide. In 2023, the American Academy of Pediatrics (AAP) developed the blueprint for youth suicide prevention, which calls for universal yearly suicide risk screening for adolescents ages 12 years and up during preventative wellness visits, and suicide risk screening for youth ages 8–11 years when they present with mental health concerns [2,3].

Notably, primary care (PC) is a widely accessed healthcare setting for young people, positioning it as a key site for potential suicide prevention efforts [2,4–8]. Assessing suicidality has demonstrated promise in identifying individuals at risk for suicide, and thus serves as an important step in the suicide care continuum [9–11]. Consequently, in line with the AAP’s recommendations, many medical settings, including PC, have begun to implement routine suicide screening protocols [2,12]. Several tools have been used for suicide risk screening in PC, including the Ask Suicide Screening Questions (ASQ), the Columbia-Suicide Severity Rating Scale (C-SSRS), and items on standard depression screeners that ask about thoughts of suicide and self-harm (e.g., Patient Health Questionnaire item 9) [13–15]. Such suicide prevention efforts rely on patients’ honest disclosure of STB. However, discussing STB can feel intimidating and requires patients to feel comfortable discussing STB with their provider. Thus, understanding how patients feel about being asked about STB is imperative to determine if these screening pathways are effective [16].

Previous research suggests PC providers and medical staff find suicide screening to be important and acceptable [17]. However, studies also note significant barriers to implementing routine suicide screening, including time constraints, disruptions to clinic workflow, and fears surrounding lack of training and ability to adequately support patients with STB [17,18]. While research examining patient and family attitudes regarding suicide screening is more limited, studies to date suggest that adult PC patients find routine depression screening using the PHQ-9 to assess suicide risk important and acceptable [19]. Emerging research with younger populations suggests that adolescent and young adult PC patients and their caregivers find suicide screening using the ASQ in both PC and the Emergency Department (ED) to be important, acceptable, and feasible [20,21].

Existing studies also highlight potential challenges that may impact how patients respond on suicide screeners [19,21], and indicate that screeners may not always accurately capture patients’ STB. For example, Simon and colleagues (2013) found that one fourth of adult patients who attempted suicide a week after completing the PHQ-9 responded “Not at all” to the ninth item (Simon et al., 2013), indicating no suicide risk [10]. Similarly, Fox and colleagues (2022) found that many adolescents are hesitant to disclose STB to medical providers, and Wyllie and colleagues (2025) suggest this may be due to mental health stigma [22,23]. Research highlights the importance of screening questions feeling relevant to patients’ experiences because if a screener does not ask about a specific feeling or experience, patients may not disclose it [24].

Given the growing emphasis on adolescent suicide screening in PC and the lack of insight into how adolescent’s perceive screening practices, the current study aimed to understand adolescents’ perspectives on suicide risk screening in PC. This research is critical to developing a better understanding of what contributes to adolescents either disclosing or avoiding disclosure of STB to their doctors. Results from this study can be used to inform adaptations to suicide risk screening and assessment practices to better equip healthcare settings to prevent youth suicide.

## Methods

### Ethics statement

All study procedures were approved by the University of Washington Institutional Review Board [STUDY00019327]. Assent/consent/parent permission forms were provided to all prospective participants. A trained research staff member met with prospective participants to verbally review consent documents. Following reviewing the document together, participants were given an opportunity to ask questions and read through the document on their own. Research staff answered any questions and confirmed participant understanding prior to obtaining consent. All participants provided informed verbal assent or consent prior to study participation. For participants under the age of 18 years, parents/legal guardians also provided verbal parent permission for their child to participate in the study prior to study participation. The IRB approved all verbal assent/consent/parent permission procedures. Research staff conducting consent documented informed assent/consent/parent permission including: date of assent/consent/parent permission, research team member conducting consent, and confirmation that (1) the assent/consent/parent permission document was provided to the participant, (2) the participant reported understanding of the study, (3) the participant’s questions were answered, and (4) the participant provided verbal assent/consent/parent permission to participate in the study. Documentation for youth (under age 18 years) who assented to participate also included the date that the parent permission form was provided to parents and documentation of if the parent provided verbal permission for their child to participate. If parent permission was given, the date parent permission was obtained and the research staff member who reviewed the parent permission form with the parent was also documented. Information regarding the voluntary nature of the study, information about confidentiality and privacy, and information regarding how to withdraw from the study were provided as part of informed assent/consent/parent permission.

### Setting

Suicide screening practices were observed at three PC sites – one urban, one suburban, and one rural. While all clinics implemented some version of mental health and suicide screening yearly at well-child visits, specific suicide screening practices differed across sites. Suicide screening ranged from paper and pencil questionnaires to self-report questionnaires on an iPad. All clinics administered screeners in the waiting room. Two clinics started suicide screening at age 12, while one clinic started at age 11. One clinic used the 9^th^ item of the PHQ-9 (“thoughts that you would be better off dead or of hurting yourself in some way”) [15] to assess suicide risk, and two sites had patients complete both the PHQ-9 and Ask Suicide Questionnaire (ASQ) [13]. At one site, any patient who endorsed suicide risk on the PHQ-9 or ASQ were then followed up with using the C-SSRS [14].

### Participants and recruitment

Adolescents were recruited from outpatient healthcare clinics in rural, urban, and suburban Washington State to participate in the study between August 20, 2024 and March 14, 2025. Participants were eligible if they were 10–18 years old, spoke fluent English, saw their pediatrician at least once a year, and had a self-reported history of STB. Adolescents were eligible if they self-reported either past of current STB including both passive and active suicidality. Adolescents were not eligible to participate if they were in a current suicidal crisis (assessed via the ASQ and the C-SSRS) [13,14] of if they reported a suicide attempt or plan/intent within the past 3 months. Study recruitment flyers were posted in clinic waiting rooms and shared with patients by clinic staff. Interested individuals were invited to complete a brief online interest survey that was used to assess basic demographic information (e.g., age, gender, race/ethnicity, geographic location) and inclusion criteria. A purposive sampling approach was used to include similar numbers of participants from urban, suburban, and rural PC settings. Eligible individuals were then invited to participate in the study.

Twenty 12–18-year-old adolescents participated. Participants provided verbal assent or consent and all participants under age 18 also had a parent or legal guardian who provided verbal consent for them to participate. Following consent procedures, all adolescent participants were administered the ASQ by a trained research staff member to assess current suicide risk. [13] Participants who endorsed “yes” to any item on the ASQ were also administered items from the C-SSRS to gain a better understanding of risk.^14^ No participants who completed suicide screeners endorsed current suicidal plan or intent. Thus, all participants who consented were eligible to participate in the study.

Following suicide risk screening, participants completed an interview and self-report measures. Participants were offered a $30 - $50 gift card or school volunteer credit for participating.

### Measures

#### Semi-structured Interview.

Participants completed a semi-structured interview over Zoom conducted by a research staff member trained in qualitative interviewing techniques. Each interview was audio recorded and transcribed. Questions focused on participant perspectives and experiences with suicide care in PC. The interview was broken into 6 overarching topics: (1) general healthcare experience in PC, (2) mental health and suicide discussions in PC, (3) support wants and needs, (4) barriers to care, (5) facilitators, and (6) recomendations for improvement. The current analysis focuses on questions related to suicide screening and assessment. Please see S1 Table for the adolescent interview guide.

#### Demographics questionnaire.

Participants completed a more thorough self-report demographics questionnaire that expanded on items from the screener survey and included items asking about geographic location (urban/suburban/rural), age, gender, race, ethnicity, mental health and treatment history, and family socioeconomic status.

#### Child mental health.

Anxiety was measured using the **Generalized Anxiety Disorder Scale-7 (GAD-7**) [25,26]. The GAD-7 is a frequently used self-report screener of anxiety that assesses the frequency of anxiety symptoms in the past 2 weeks on a 4-point scale from “Not at all” to “Nearly every day.” Depression was assessed using the **Patient Health Questionnaire-9 (PHQ-9**) [15]. Participants self-reported the frequency of depression symptoms over the past 2 weeks on the PHQ-9 using a 4-point scale ranging from “Not at all” to “Nearly every day”.

### Analyses

Descriptive statistics were calculated in SPSS version 28.0 (IBM SPSS, 2024) to characterize participant-reported demographics, anxiety, and depression. Each interview transcript was reviewed by a research team member for accuracy (SD, AA, AH). Following this, interview transcripts were uploaded into Dedoose and analyzed by members of the research team (SD, AA, AH) using a combination of deductive and inductive thematic analysis to identify new information about user needs, barriers, and facilitators to engaging in suicide screening and assessment [27,28]. A preliminary codebook based on the consolidated framework for implementation research (CFIR) domains [29] was used alongside codes based on topical areas from the interview guide. All transcripts were double coded and coders met weekly to review and add codes and to resolve any disagreements through a consensus process until no discrepancies emerged. Codes were iteratively added and refined until thematic saturation was reached. Coders organized codes into themes which were iteratively reviewed and refined with all team members to ensure agreement and were compared across the different geographic settings.

## Results

### Participants

Twenty adolescents (M_age_ = 15.55 years, SD = 1.82 years) participated in the study. Approximately 40% of participants were seen in an urban PC clinic, 30% were seen in a suburban clinic, and 30% were seen in a rural clinic. Approximately half (45%) identified as female. Nineteen (95%) participants reported having ever received mental health treatment and 14 (73.7%) reported they were currently receiving mental health treatment. All participants reported they had talked to their PC provider about mental health at some point. See Table 1 for additional demographic information.

**Table 1:** Participant Demographic Information.

	Teens
Mean Age (sd)	15.55 (1.82); range 12–18 years
Mean PHQ-9 Score (sd)	11.05 (7.55)
Mean GAD-7 Score (sd)	9.10 (5.04)
ASQ “ever tried to kill yourself?”	10 (50%)
Geographic setting of primary care clinic	
Urban	8 (40%)
Suburban	6 (30%)
Rural	6 (30%)
Self-Reported Gender	
Male	6 (30%)
Female	9 (45%)
Other Gender	5 (25%)
Race/Ethnicity*	
American Indian/Alaska Native	1 (5%)
Asian	0 (0%)
Black or African American	2 (10%)
Hispanic or Latinx	2 (10%)
Hawaiian or Pacific Islander	2 (10%)
White	20 (100%)
Mental Health History	
Anxiety diagnosis	17 (85%)
Depression diagnosis	17 (85%)
ADHD diagnosis	11 (55%)
Other psychiatric diagnosis	8 (40%)
“I do not know” or Prefer not to say	2 (10%)
How much money does your family have?	
“Just enough to get by”	1 (5%)
“We only have to worry about money for fun or extras”	9 (45%)
“We never have to worry about money”	7 (35%)
Don’t know or prefer not to say	3 (15%)
“Are you currently receiving mental health treatment?"	
Yes	14 (73.7%)
No	4 (21.1%)
Prefer not to say	1 (5.3%)
"Have you ever received mental health treatment?”	
Yes	19 (95%)
No	1 (5%)
“Have you ever talked to your primary care provider about mental health?”	
Yes	20 (100%)
No	0 (0%)

*Participants could endorse more than one race/ethnicity.

### Themes

We identified 5 themes that reflect participants’ perceptions of suicide screening in PC. Themes were observed across all 3 geographic settings. Results did not differ across settings, and therefore, are discussed together. See S2 Table for additional representative quotes.

#### Theme 1: Appropriateness of being asked about STB.

Participants think being asked about STB in PC is important and shows that providers care. Nearly all participants reported they either felt comfortable with or thought being asked about STB at their PC visit was important. One participant shared, “I actually don’t mind it, and it shows that like they care about mental health.” Other participants expressed that being asked about STB helped them self-reflect and discuss topics they might not have brought up otherwise, “It’s very comfortable. I’m really glad that she does it honestly because she asks a lot of questions that I feel wouldn’t come up, like I wouldn’t think about if I had to be the one running the conversation.”

Even when participants voiced discomfort with being asked about STB, they often shared that STB screening was an important step to getting help. For example, one participant shared, “I would say it’s not always fun. It’s kind of annoying having to tell them how you feel. Then you’re debating whether you should lie or not. And then it’s very hard to do. But I think once you tell ‘em [and] when you get the right help, it definitely helps you a lot...”

#### Theme 2: Impact of the language and delivery of suicide risk screeners on adolescent STB disclosure.

Language in suicide risk screeners and how questions about STB are asked impacted participants’ comfort and honesty in disclosing STB. Notably, participants highlighted that how questions about STB are asked impacts how adolescents respond. Although participants were mixed on if they preferred to be asked about STB via a self-report screener or verbally by their doctor, many participants shared that the tone and language used in STB screening questions often comes across as too “direct”, “blatant” or “pushy”, which participants found scary and confusing and sometimes made them less likely to respond honestly. Additionally, participants shared that the order of questions matters, “…sometimes [questions about mental health and STB] it comes off a bit strong. I think they should ask you questions about your history with things first.”

Some participants also noted that the questions and response options were often confusing and difficult to answer. One participant stated, “‘Are you thinking about hurting yourself?’ could be so many different things, like self-harm versus killing yourself.” Another participant shared, “the questions are usually ‘yes’/’no’ answers, or ‘not likely at all’ to ‘always’...and that’s a very weird scale.” Participants also shared that, even though being asked about STB is important, answering the same questions at every visit feels overwhelming. Some participants expressed that asking certain questions during the screening process could also be harmful, “... I know for a few questions like, ‘how did you do this?’ ‘Have you ever done this in your life?’ It could bring back memories that they don’t exactly want to think of... if it didn’t ask *how* you did it and they just asked if you *ever* did that - I think that can also get more honest answers, instead of making somebody remember how they felt during that time.”

Additionally, participants reported concerns regarding confidentiality when filling out these screeners in spaces where other people might see their answers (e.g., the clinic waiting room). One participant shared, “... if it was not a confidential space, if you had other people in there or if it was in the lobby, that wouldn’t be good, but if it was just you and your doctor, it’d probably be fine.” Another shared, “Usually one of my parents will bring me there and they’re there with me. They’re usually sitting next to me, and sometimes I’m like, I don’t want them to see it [the STB screeners] either.”

Finally, participants suggested that when verbally asked about STB, doctors should be more conversational and engaging so that patients feel heard, “…it’s easier if they build more of a conversation and include the questions in it casually and not just checking off questions off the list...” Participants also shared that it is important for doctors to ask more contextual and “open-ended questions instead of that screener” to help adolescents feel more comfortable and willing to be honest. Participants shared several examples of how they want to be asked about STB, including “‘Hey, how are you doing today?’... ‘How’s school going?’…‘Have you had any tests recently?’ …even just small things like that to break the barrier down of, ‘Okay, I’m going to actually think about how I’m doing’ is a really good first step to making people more comfortable with the idea of opening up.”

#### Theme 3: Impact of provider-patient interactions and relationships on STB disclosure.

Provider-patient relationships and interactions impacted participants’ comfort and honesty in disclosing STB. Many participants noted how relationships and interactions with doctors directly impacted the likelihood they would disclose STB or other mental health concerns. Participants reported that provider behaviors that were perceived as lacking genuine engagement or caring, such as focusing on taking notes instead of engaging in open conversations, made it harder to share STB with them. Several participants commented on tone of voice, facial expression, and attitude. One expressed, “I just feel like they feel very stiff to me. They’re very scary.” Multiple participants reported that providers who seemed uncomfortable or reacted strongly to STB made the experience scary and decreased comfort with disclosing STB. Ultimately, participants shared that these interactions could be improved by providers “Be[ing] gentle about it. Don’t start asking a flurry of questions. Be very kind about it and just understanding about it.”

In addition to individual interactions related to discussing STB, several participants shared that their relationships with their providers impacted their willingness to respond honestly about mental health and STB. Participants who had longer-term and trusting relationships with their providers reported feeling more comfortable discussing STB with them. In contrast, not having an established relationship with their provider was seen as a barrier to endorsing STB.

#### Theme 4: Impact of mental health stigma on STB disclosure.

Participants often did not feel comfortable disclosing STB due to perceived mental health stigma, even if they thought being asked about STB in PC was important. Participants shared that “the stigma of being able to even bring that [STB] up in the first place [is tough] because like, not a lot of people talk about it... basically nobody does.” Other participants expanded that, “I think it’s just this idea that suicide is this big scary thing and it’s really scary to talk about and it’s really hard to talk about, and it’s kind of like hushed…the stigma around it makes it hard to want to reach out. It makes it hard when someone reaches out to you.” Ultimately, participants shared that this stigma led to feelings of guilt, embarrassment, and shame around STB that make it difficult to tell doctors and ask for help. In line with this, many participants voiced that they wished that mental health and suicide conversations could be more normalized, which would make it easier to be honest and seek help, “People need to talk about it. And because nobody talks about it, it’s hard for teens to know what to do and that doesn’t help anything.”

#### Theme 5: Fear of how others, especially caregivers, may react and potential consequences of disclosing STB (e.g., loss of autonomy).

Participants feared how others, especially parents or caregivers, would react and what consequences there would be if they disclosed STB. For example, one participant shared “I think a lot of it is fear of what are people going to think of me... I don’t want to feel judgment. I don’t want to feel like I’m doing something wrong by how I’m thinking and how I’m feeling.” In particular, participants shared concerns about how parents would react to them, “I think it [STB] is kind of hard to, I guess, talk about with parents because I’ve had so many friends who would kind of bring it up to their parents and their parents would brush it aside or be like, I don’t know, ‘Just get over it’.”

Participants also expressed that not understanding confidentiality and what doctors are obligated to tell parents led to lack of disclosure on screeners because they often fear how caregivers will react. In line with this, participants felt that having parents present when being asked about STB would make them less likely to disclose and highlighted that adolescents should be asked about mental health without parents present, “[Asking about STB is] a good idea as long as the parents get removed from the room because, again, that’s very uncomfortable in front of parents,” and, “I would be more likely to be honest if my parents aren’t in the room.”

Several participants expressed that honesty about STB was dependent on adolescent-parent relationships. Many voiced concerns related to parents getting upset or punishing adolescents for having STB or talking about mental health and fears that sharing their STB would lead parents to be sad, upset, or worried, or cause them to direct their distress towards their child. For example, one participant shared, “…usually on the suicidal one [screener question], I tone down a little more … it’s more just for me personally that I don’t want my parents to be sad or just feel upset, or anything. I don’t want to make them sad with anything going on with me.” In general, participants thought this was a common sentiment amongst adolescents, “I feel like a lot of ‘em [adolescents] are just more worried that their parents are going to get upset at them, so then they don’t want to talk about it [STB] or reach out or anything like that.” Another participant discussed how parents might also be contributing to adolescents’ mental health struggles, “A lot of times the parent can be part of what brings on any mental health issue. A lot of it can be brought on by parents or your parents don’t believe you or whatever. So then when your parent’s in there, it’s like, well, now I don’t want to say anything.”

Uncertainty about what would happen or fear of repercussions (such as loss of autonomy) after sharing STB was also reported as a common fear amongst participants, “You don’t want to answer honestly all the time because you’re already like, ‘Okay, I’m angry, I’m obviously troubled, and now I’m going to answer questions and you’re probably going to send me away or force me into some form of treatment that people might not always want to do’ and I think it just freaks people out.” Participants reported that if doctors gave adolescents more of a role in treatment decision making, that would make the process feel easier. They suggested that providers “...respond in a more caring tone, like, okay, here’s your options... So they [patients] have choices on how they want to take care of this instead of feeling like they need to go through this one path or they’re not gonna ever feel better.”

Several participants suggested that helping adolescents understand what they will be asked in advance and helping them understand their rights to confidentiality would help with STB disclosure. Additionally, participants highlighted the importance of working with adolescents to determine if their parent is a person that can support them or if they need to work with a different trusted adult to support their safety. Participants felt that addressing these points of discomfort could increase honesty and likelihood of adolescents disclosing STB in PC.

## Discussion

This is among the first studies to our knowledge to ask adolescents about their perspectives on being screened for suicidality at their PC doctor’s appointments. Other studies that have asked pediatric patients for their perspectives on suicide risk screening have either taken place in ED settings or specifically examined young people’s feedback to receiving the ASQ in a single PC system [20,21]. The current study identified 5 themes that extend findings to broader pediatric PC settings [19].

Similar to previous studies (e.g., Horowitz et al., 2022; Richards et al., 2019), participants agreed that regularly screening for STB and mental health concerns in PC is important and that PC is an appropriate setting to ask about STB because it helps normalize the conversation, shows providers care about mental health, aids in self-reflection, and helps patients obtain mental health support if they need it [19,20]. Notably, findings were consistent across geographic settings, suggesting adolescents’ openness to suicide screening may transcend community or region-specific stigma surrounding suicide and mental health. However, similar to previous investigations, while participants agreed that asking about STB in PC is important, many shared concerns with how STB is asked about. In particular, participants shared concerns with the directness and framing of screening questions, impacts of negative interactions with clinicians during verbal assessments, mental health stigma, fear of parents/caregivers finding out, and fear of losing autonomy after disclosure [19,21].

Participants had several suggestions to improve screening processes, many of which are likely feasible to implement in PC. First, similar to findings from Cafferty and colleagues (2025) in the ED, participants suggested that screeners would be improved if patients first received information explaining confidentiality and why adolescents are asked about STB [21]. Additionally, in line with Cafferty and colleagues (2025), participants highlighted the importance of being able to answer these questions privately [21]. However, while Cafferty and colleagues (2025) found that adolescents did not have a preference regarding where they completed electronic screeners, results from the current study found that adolescents generally preferred *not* to complete suicide screeners in the waiting room because of the proximity to parents or other patients resulting in a lack of privacy. These results may differ due to differences in PC versus ED settings. For example, parents are often aware of their child’s STB by the time adolescents are seen in the ED and therefore, adolescents may have fewer concerns about privacy while answering suicide screeners in ED settings. Additionally, how information is shared between patients and caregivers in the ED may differ compared to how it is shared in PC. There may also be differences regarding if screeners are completed electronically versus using paper and pencil, and differences in waiting room sizes or layouts may also impact perceived privacy.

Similar to feedback from using the PHQ-9 item 9 to assess suicide risk with adults [19], participants frequently endorsed difficulties with the language used in suicide risk screeners. Participants in our study received a variety of different screeners across their respective clinics, including the PHQ-9 (using item 9 to assess suicide risk), the ASQ, and the C-SSRS. However, across clinic settings, participants noted feeling uncomfortable with the direct language that suicide screeners use, as well as challenges with knowing how to respond based on the “yes”/“no” or Likert scale options. Participants frequently suggested that their doctor’s offices needed to incorporate more holistic approaches to mental health and suicide risk questionnaires, including adding questions that ask more broadly about general updates in their life and what may be going well for them. Together, results suggest that suicide screening practices could be improved by including more open-ended and conversational questions and starting assessments by asking broader questions about life updates prior to moving into questions about STB. Gathering additional input from a broader group of adolescents to help shape the language and order of questions, to promote acceptability, and to increase comfort and honesty in responding is needed.

Participants overwhelmingly agreed that having a long term and trusting relationship with their provider is paramount to being open and honest about their mental health. However, how providers communicate also plays a critical role in how adolescents respond to questions related to STB. In particular, participants highlighted the importance of providers showing they are actively listening and engaged in conversations by expressing genuineness and empathy, not taking notes during the conversation, and not reading questions off of a list or responding in a negative tone. These results support previous research investigating suicide risk disclosure that highlights positive relationships and compassionate, nonjudgemental interactions with PC providers as factors that increase the likelihood of disclosure [19,30]. These findings point to a need for additional training for PC providers to improve patient-provider interactions during suicide risk assessment to enhance patient comfort.

Other barriers to endorsing STB were mental health stigma, fears of how parents would react, and losing autonomy. Previous research has found all of these to be important factors a person considers when determining whether or not to disclose STB [18,19,22,31–35]. However, contrary to the thought that mental health stigma would be a greater barrier for adolescents in more rural settings [18], we did not find any differences in reported mental health stigma impacting STB disclosure across the different geographic settings. Instead, we found that stigma was one of the most reported barriers across all settings, suggesting that even in larger communities where mental health resources are more accessible, adolescents still feel the impacts of mental health stigma. Future research should use a mixed methods approach to gain a more in depth understanding of what stigma looks like in different contexts and how it plays a role in STB disclosure across communities.

Concerns specific to adolescents, such as fear of doctors telling their parents about mental health concerns, and how their parents will react are well known [22,36]. Despite these challenges, little has been done to actively address them to improve mental health and STB disclosure among adolescents. Similar to Cafferty and colleagues (2025), several participants suggested that more proactive psychoeducation for both adolescents and caregivers regarding why adolescents are asked about STB, the limits of confidentiality, and patients’ rights to choose their care would be useful in aiding more honest disclosure.

Additional adaptations to suicide prevention practices in PC should consider: (1) providing information regarding screening to patients and caregivers in advance, (2) providing psychoeducation about mental health and suicide to all adolescent patients and their caregivers to help normalize mental health discussions, (3) providing private spaces to complete self-report screeners, (4) asking broader questions and gathering information about social and emotional functioning, including what is going well, before asking about mental health symptoms and STB, (5) using active listening, displaying interest, validation, and empathy without judgement and responding to patient disclosure of STB in a calm manner, (6) using patient-centered language when assessing mental health and STB, and (7) promoting patient autonomy as much as possible in treatment decision making and decisions regarding how to include their caregiver in conversations about STB. However, research in this area is lacking and is a critical area for future investigation.

## Limitations and future directions

While this study provides important insight into adolescent’s views on suicide risk screening in PC, there are several limitations. First, while a strength of the current study is the recruitment of adolescents from urban, suburban, and rural settings, it is important to acknowledge that all recruitment occurred in Washington State and included a largely White participant population. Additionally, participation in the current study required adolescents under the age of 18 years to have parent permission to participate. By nature, this suggests that parents were aware, at least to some extent, of their child’s STB history, and that adolescents were comfortable communicating about their study participation with their parents. In contrast, adolescents who did not want their parents to know about their STB may have chosen not to pursue participation in this study.

Conducting research with minors typically requires parent consent. However, requiring parent consent can unintentionally result in samples that may underrepresent adolescents with less family support and poorer family relationships. This pattern has been observed in other sensitive and stigmatized research areas, and can affect the generalizability of findings, which ultimately impacts interventions [37,38]. Thus, future work is needed to allow more diverse adolescent perspectives to be included. Potential avenues to achieve this include consideration of waivers of parental consent for mature minors and confidential recruitment pathways, when appropriate. These approaches may allow for privacy while still maintaining protections for participant safety and well-being, and would ultimately promote inclusion of more diverse adolescent perspectives.

Notably, the current study includes only adolescent perspectives, and only included adolescents with lived experience of STB. While adolescents reported varied background and mental health symptoms, and many adolescents reflected on the first time they discussed STB with their healthcare provider, by only including adolescents with STB the perspectives shared in this study may differ from those of adolescents without STB, or those identified with STB for the first time, who potentially have less familiarity with suicide risk screeners and conversations. Despite this, adolescents in the current study highlighted several challenges to discussing and disclosing suicide risk in PC, highlighting important areas for advancement. Notably, the goal of this qualitative study was to gain an in depth understanding of how adolescents with STB view suicide screening practices in PC settings. While qualitative research is not meant to be generalizable, additional research is needed to investigate and integrate findings from adolescents from other racial/ethnic groups and communities, as well as adolescents with and without mental health and STB histories, adolescents identifying STB for the first time, and adolescents with varying levels of parent support. It will also be important to take provider perspectives into account to develop tools and frameworks that are both feasible to implement in PC and acceptable to patients and families.

## Conclusions

Assessing for suicide risk in PC remains a promising suicide prevention strategy [2,3]. However, suicide risk screening relies on adolescents disclosing STB. Without honest disclosure, existing suicide screening practices are ineffective. Thus, there is a critical need to improve suicide screening and assessment to be more acceptable to the individuals who receive them. Participants in the current study shared their perceptions of current screening practices and highlighted several avenues for improvement that can be delivered on a universal level, including developing and administering universal prevention materials with information on suicide, mental health, screening practices, and confidentiality to families seen in PC. Future research should use co-design methods with diverse adolescents, PC staff, and parents/caregivers, to improve current suicide screening pathways to enhance acceptability and appropriateness of these materials while ensuring feasibility and effectiveness in PC settings.

## Supporting information

S1 Table Adolescent Interview Guide. (DOCX)

S2 Table Additional Representative Quotes and Adaptations Considerations. (DOCX)
