# Supplementary material for: Understanding adolescents’ perspectives on suicide risk screening in primary care: Implications and insights for implementation and practice
Source: PLOS Ment Health. 2026 May 7;3(5):e0000607. doi: 10.1371/journal.pmen.0000607 (PMC13152160; doi:10.1371/journal.pmen.0000607)
Supplement: S1 Table — (DOCX) [file pmen.0000607.s001.docx]

**S1 Table. Adolescent Interview Guide**

| **Topic** | **Interview questions** |
| --- | --- |
| **General Health Care Experience** | - Can you tell me about your experience when visiting your primary care provider (like a doctor or nurse)? (For example, how often do you go? Do you always see the same or a different doctor? Do your parents come in with you? How comfortable are you with these providers)? |
| **Mental Health and Suicide Discussion** | - Has your doctor ever asked you about if you’ve had any suicidal thoughts or behaviors (STB)?   - If yes, how were you asked about it? (e.g., doctor asked me verbally/I completed a screener in the waiting room, etc.)   - Did they ask you about any other mental health problems?   - Who (if anyone) followed up and talked to you about these thoughts and how you were feeling?   - How did you feel about being asked these questions?     - Overall, what parts went well/did you like?     - What didn’t go well/did you not like? - Was your parent involved?   - If so, how were they involved and how did you feel about their level of involvement? - If kids/teens are having STB, who or where do you think they would go to get help? - How would you feel if your doctor/primary care team asked you about if you were having any STB every year during your regular/yearly check-ups, even if you didn’t tell them you were having STB before?   - What concerns, if any, might you have about this?   - How comfortable would you feel telling your doctor/primary care team if you were having STB?   - When being asked about STB, do you think kids and teens would prefer to talk to the doctor by themselves or to have a parent/caregiver with them?     - How do you think kids and teens would prefer that their doctor ask them about STB? For example, would you prefer your doctor verbally ask you about it when they’re in the room with you, or have you complete a screener (like on paper or an iPad) before coming into the exam room?       - If you’d prefer your provider ask you about STB – would it matter who asked you these questions (your main doctor, the nurse, the person rooming you, etc.)?       - Would you prefer to answer these questions with or without a parent/caregiver present?   - How do you think kids/teens would want their doctor to respond if they were having thoughts of suicide? - How would you feel if your parent/caregiver talked to your PCP if they were concerned about you experiencing STB? How do you think other kids/teens would feel? - What are the most important things that you think a doctor or other healthcare provider should discuss with kids/teens when it comes to mental health and suicide in primary care settings?   - What do you think are the main things they should discuss with parents/caregivers? - What would you think about having to stay longer at the appointment to address mental health or STB concerns versus going home and having someone follow up with you over Zoom/phone?   - What feels comfortable/uncomfortable?   - How long do you feel it is ok to wait for someone to follow up with you if you were to report STB concerns to someone at your doctor appointment? |
| **Support Needs** | - If you were having STB, what do you think would help? - What kind of support do you think kids/teens need when talking about mental health and suicide at their doctor’s office? - Are there any specific types of services or resources you think should be available to help kids/teens who are struggling with suicidal thoughts? What kind? |
| **Exploring Wants** | - If you were designing an intervention for kids and teens with thoughts of suicide in primary care, what would it look like? - What role do you think your doctor, other primary care staff, and other friends, family, or other trusted people should play in assessment and treatment of STB? |
| **Barriers** | - What are some of the challenges you think kids/teens face when trying to get help for STB? - What do you think makes it hard for you or other teens to talk about STB with a doctor or nurse? - Have you ever avoided discussing your mental health with a healthcare provider or weren’t honest when asked about STB so you didn’t have to talk about it with them?   - If so, why? - Was there anything negative about these experiences [talking to doctor about STB]? - Do you have any ideas for how we could help overcome these challenges? |
| **Facilitators** | - Can you share any positive experiences you've had discussing mental health or STB with a healthcare provider? - What made those experiences positive for you? |
| **Recommendations  for Improvement** | - What changes do you think could make it easier for kids/teens to talk about these concerns in primary care? - Do you think there’s any reason for schools, community programs, or online resources to partner with primary care to support kids and teens’ mental health and/or help manage STB?   - If yes, how would you want them to be involved? - How can healthcare providers better support kids and teens when talking about mental health and suicide? - What role do you think parents/caregivers should have in suicide care for kids/teens? - What is the best way for a healthcare provider to communicate with you – or how would you want to be able to communicate with your provider if you wanted to talk to them? (ex, through your medical record messaging system, phone call, text, email, etc.) |
| **Final Thoughts** | - Is there anything else you think is needed for primary care-based STB services for teens? - Is there anything else you’d like to share that we haven’t discussed? |
