# Supplementary material for: Understanding adolescents’ perspectives on suicide risk screening in primary care: Implications and insights for implementation and practice
Source: PLOS Ment Health. 2026 May 7;3(5):e0000607. doi: 10.1371/journal.pmen.0000607 (PMC13152160; doi:10.1371/journal.pmen.0000607)
Supplement: S2 Table — (DOCX) [file pmen.0000607.s002.docx]

**S2 Table.** Additional Representative Quotes and Adaptation Considerations

| **Theme** | **Representative Quotes** | **Current State** | **Proposed Adaptations** |
| --- | --- | --- | --- |
| **Theme 1:** Participants think being asked about STB in PC is important and shows that providers care | *“I don't mind. Like I know it's for a good reason. Sometimes it's like I'll be having a really bad day and I really don't want to answer it. Yeah, it's not the worst thing. And if it helps them like know me, it helps them treat me or whatever, then I guess it's good.”*    *“I don't see it [suicide screening] as a bad thing. I think it's kind of a good thing.”* | - Inconsistent suicide screening, with some clinics using universal screening approaches and others only assessing suicide risk if other mental health concerns arise - Age of suicide screening initiation is variable - Primary mode of suicide screening is done via standardized self-report screeners - Inconsistent provider follow-up assessment | - Build standardized pathways that incorporate asking patients about mental health and suicidality that involve at least some verbal assessment and follow-up |
| **Theme 2:** Language in suicide risk screeners and how questions about STB are asked impacted participant’s comfort and honesty in disclosing STB | *“I think that it's the words that they use that make it [talking about STB] a little bit more intimidating.”*  *“They just say it in a monotone way just to say it …if they're going to ask you and talk to you about it, I feel like they need to actually care and not just say it as a way to move on to the next question or to say that they said it. I think that especially in a topic of suicide, how invested a person is when talking to somebody about it really shows a lot. And it also determines how much the other person, how much they want to open up and talk about it.”* | - Implementation of standardized screeners including the ASQ, C-SSRS, or PHQ-9 (item 9) (either as self-report or verbally) | - Provide information prior to screening so teens know why they’re being asked about STB - Use patient-centered language, reflecting the words patients use to describe STB - Start with asking broader questions and gathering information about social and emotional functioning, including what is going well - Display interest, validation, and empathy without judgement |
| **Theme 3:** Provider-patient relationships and interactions impacted participant’s comfort and honesty in disclosing STB | *“...it was the fact that it was just this stone face, ‘do you want to kill yourself?’ I was like, girl, can we maybe have a little more compassion?”*  *“... I think if the doctor and I would have just a conversation without any, you know, notes being taken or being typed out onto a computer, I think that would definitely be helpful ‘cause then I could feel like I was just talking to someone about it and not being surveyed on my suicidal thoughts.”*  *“A lot of doctors, if you mention having a plan or anything like that, they’ll get kind of intense. I remember getting super freaked out once when I was a little younger. I said something like, I’m having thoughts of killing myself and the doctor, I don’t know, gave me a look. And I was like, wait, never mind, never mind. And the doctor was like, you have to tell me those things.”* | - Variable patient-provider relationships and interactions regarding conversations about mental health and STB | - Respond to STB in a calm manner, express partnership with patient to help them get support to feel better - Use active listening - Display interest, validation, and empathy without judgement - Do not take notes while having conversations about STB with patients |
| **Theme 4:** Participants often did not feel comfortable disclosing STB due to perceived mental health stigma | *“I wish we could normalize the actual conversation about how kids are feeling. I mean, even just from a young age to destigmatize the idea of just talking about emotions, period being like, no, I don't want to kill myself, but I'm having a tough day. I want that to be normal.”*  *“I was embarrassed talking about it and just feeling annoyed that you have to talk about it. You don't want to talk about it, but you also want help ...”* | - Mental health stigma exists amongst providers, patients, families, and communities - Variable amounts of discussion about mental health and STB within families, patient-provider relationships, etc. | - Universal psychoeducation to patients and parents to help normalize mental health discussions and provide psychoeducation - Universal psychoeducation for parents to reduce mental health stigma and promote discussion about mental health with their children - Training for providers to reduce stigma |
| **Theme 5:** Participants feared how others, especially caregivers, would react and what consequences there would be if they disclosed STB | *“I also was afraid, I'm like, if I say anything, then they're going to tell my parents and I have to deal with my parents about it and all that.”*  *“I know a lot of kids also worry that if they say they have them [STB], they're going to end up put under holds or being admitted to the mental health hospital because nobody really wants that.”* | - Inconsistent practices around asking parents to leave the room during conversations about mental health - Inconsistent practices regarding sending patients to the ED if STB is endorsed - Adolescents frequently lack understanding regarding confidentiality | - Psychoeducation provided to all adolescents and caregivers regarding confidentiality - Training for providers on least restrictive care model/zero suicide framework - Assess risk/benefit of bringing caregiver into conversation - Ask patient alone if they want caregiver in the room or not during mental health/STB conversations - If giving self-report screeners, provide space away from caregivers for patients to be able to fill out screeners - Promote patient autonomy (as much as clinically appropriate) in treatment decision making |
